# Long-Term Oncologic Outcomes of Omitting Axillary Surgery in Breast Cancer Patients with Chest Wall Recurrence after Mastectomy

**DOI:** 10.3390/cancers16152699

**Published:** 2024-07-29

**Authors:** Geok Hoon Lim, Veronica Siton Alcantara, John Carson Allen, Seyed Ehsan Saffari, Veronique Kiak Mien Tan, Kiat Tee Benita Tan, Sabrina Ngaserin, Su Ming Tan, Lester Chee Hao Leong, Fuh Yong Wong

**Affiliations:** 1 Breast Department, KK Women’s and Children’s Hospital, Singapore 229899, Singapore; veronica.siton.alcantara@kkh.com.sg; 2 Duke-NUS Medical School, National University of Singapore, Singapore 169857, Singapore; 3 SingHealth Duke-NUS Breast Centre, Singapore 168582, Singaporesabrina.ngaserin@singhealth.com.sg (S.N.);; 4 Centre for Quantitative Medicine, Duke-NUS Medical School, National University of Singapore, Singapore 169857, Singapore; 5 Department of Breast Surgery, Singapore General Hospital, Singapore 544886, Singapore; 6 Division of Surgery and Surgical Oncology, National Cancer Centre, Singapore 168583, Singapore; 7 Breast Service, Department of Surgery, Sengkang General Hospital, Singapore 544886, Singapore; 8 Division of Breast Surgery, Changi General Hospital, Singapore 529889, Singapore; 9 Department of Diagnostic Radiology, Singapore General Hospital, Singapore 169608, Singapore; 10 Division of Radiation Oncology, National Cancer Centre, Singapore 168583, Singapore

**Keywords:** breast cancer, mastectomy, recurrence, axillary staging, sentinel lymph node biopsy

## Abstract

**Simple Summary:**

The management of the axilla for post-mastectomy breast cancer patients who develop a chest wall recurrence (CWR) remains to be established. This study aimed to determine if omitting axillary staging surgery for these patients resulted in an increased risk of second recurrence. A total of 194 patients with CWR, with a median follow-up of 59.5 (IQR 27.3–105) months, were analysed. There was no statistically significant difference in second recurrences between patients with or without axillary surgery during the excision of the CWR.

**Abstract:**

Background: The management of the axilla in breast cancer patients with isolated chest wall recurrence (CWR) after mastectomy remains controversial. Although sentinel lymph node biopsy (SLNB) for restaging is feasible, its role is unclear. We aimed to determine if the omission of axillary restaging surgery in female patients with operable presumably isolated CWRs could result in an increased risk of second recurrences. Methods: In this retrospective multicentre study, patients who developed CWRs were reviewed. We excluded patients with suspected or concomitant regional/distant metastases, bilateral cancers and patients without CWR surgery. Patients’ demographics, pathological data and subsequent recurrences were collected from a prospective database and were compared between patients with axillary lymph node dissection (ALND) and/or SLNB versus no axillary operation at CWR. Findings: A total of 194 patients with CWRs were eligible. The median age at CWR was 56.0 (IQR 47.0–67.0) years old. At recurrence, 8 (4.1%), 5 (2.6%) and 181 (93.3%) patients had ALND, SLNB and no axillary operation, respectively. Patients with no axillary surgery during CWR were associated with, at primary cancer, a lower incidence of ductal carcinoma in situ as diagnosis (*p* = 0.007) and older age (*p* = 0.022). Subsequent ipsilateral axillary (*p* = 0.768) and second recurrences (*p* = 0.061) were not statistically different between patients with and without axillary surgery at CWR on median follow-up of 59.5 (IQR 27.3–105) months. Interpretation: In patients without evidence of concomitant regional or distant metastasis at CWR diagnosis, omission of axillary restaging surgery was not associated with an increased ipsilateral axillary or second recurrences on long-term follow-up.

## 1. Introduction

Breast cancer is the commonest cancer among women worldwide and its incidence is rising globally, with a surge in breast cancer rates noted in Asia [1]. Mastectomy rates were also reportedly higher in Asia compared to their Western counterparts [2]. This could be attributed to several reasons such as the smaller breast size more frequently seen in Asian women [3], which makes breast conservation harder, late presentation of the tumour with lower screening rates [4], etc.

Despite advancements in the treatment for breast cancer, isolated locoregional breast cancer recurrences can occur at an incidence of 10–35% [5]. In patients with invasive chest wall recurrences (CWRs) after mastectomy, and no evidence of proven or suspected concomitant regional or distant metastasis at CWR diagnosis, the optimal management of the axilla in these patients remains unclear [6]. Historically, an axillary lymph node dissection (ALND) was usually carried out for axillary restaging, concurrently with the resection of the CWR, if there was no prior ALND [7]. However, ALND can be associated with many comorbidities, with the risk of arm lymphedema being four times higher [8] compared to sentinel lymph node biopsy.

Consequently, for these patients with a clinically node-negative axilla at the time of recurrence, there has been a paradigm shift from ALND to sentinel lymph node biopsy (SLNB), with ALND only being performed for patients with nodal metastasis [9]. While performing a SLNB after a mastectomy can be technically challenging, especially with a history of axillary surgery, it has been reported to be possible [10,11] in patients with CWRs.

Nonetheless, there are sparse data on the usefulness of axillary staging in this group of patients who have no evidence of concomitant or suspected metastasis elsewhere at CWR diagnosis. While the purpose of axillary staging in patients with primary breast cancer is to detect occult nodal metastasis, which can influence adjuvant treatment decisions, its role in the CWR setting has not been widely studied.

We aimed to determine if the omission of axillary staging surgery in patients with CWR and no concomitant evidence of metastasis elsewhere would result in increased second recurrences, specifically ipsilateral axillary nodal recurrences.

## 2. Materials and Methods

Stage 0-III female breast cancer patients who received treatment at five tertiary institutions in the Republic of Singapore from 1 October 1989 to 31 March 2021 were included in this retrospective multicentre study. The five institutions included the National Cancer Centre Singapore, Singapore General Hospital, KK Women’s and Children Hospital, Changi General Hospital and Sengkang General Hospital. Of this group of patients, we identified patients who had mastectomy and developed pathologically confirmed invasive CWR. We excluded patients with bilateral cancers and patients with concomitant distant and/or regional recurrences. Patients who had clinical or radiological suspicion of ipsilateral axillary nodal involvement at the time of CWR diagnosis were excluded. Patients who did not have surgery for their isolated CWRs, as well as patients who were lost to follow-up or had incomplete data, were excluded too.

In our local setting, the decision for breast-conserving surgery or mastectomy with or without reconstruction was based on patients’ tumour characteristics such as the tumour-to-breast-size ratio, presence of multiple foci/multicentric cancer, etc., and patients’ preference. Patients were discussed at a multidisciplinary tumour board meeting regarding their adjuvant treatment and were then followed up at recommended surveillance intervals after their treatment.

During each surveillance visit, a clinical examination was performed. A mammogram was performed annually for the contralateral breast. In cases when there was a suspicious mass at the mastectomy site, the patient may have undergone an ultrasound, especially if they had a post-mastectomy breast reconstruction, to further define the lesion. In some cases, an ipsilateral axillary ultrasound was performed too.

To obtain the histology of the lesion, a percutaneous biopsy or excision biopsy was carried out. If a CWR was confirmed histologically, the patient was subjected to systemic staging, usually with a CT thorax/abdomen/pelvis and bone scan. If there was no evidence of metastasis elsewhere on imaging or suspected clinically, the patient underwent a wide excision of the CWR, if it was operable. The management of the axilla in these patients with CWRs was based on their respective treating physicians’ discretion. If SLNB was performed at the time of the CWR, a blue dye and/or radioisotope was used for the localisation of the sentinel lymph node. Each patient was discussed at the multidisciplinary tumour board meeting regarding her individualised therapy. Human epidermal growth factor receptor 2 (HER2)-targeted therapy was given if indicated. Their subsequent clinic reviews followed the same surveillance schedule as for primary cancer.

Patients’ demographics, histological data and subsequent ipsilateral axillary and second recurrences, if any, were obtained from a prospectively maintained database. For the second recurrence, if there was concomitant regional/local and distant metastasis or local and regional metastasis, the patient was classified as having distant and regional metastasis, respectively. In all cases of regional and distant metastasis, evidence of ipsilateral axillary recurrence would be recorded. The collected data were then compared between patients who had received axillary surgery of either SLNB or ALND and those who had not received axillary surgery at CWR. For this study, the follow-up period was defined from the date of histological confirmation of their CWR to another recurrence or death or the last known follow-up date, whichever happened earlier.

### Statistical Analysis

Categorical variables were reported as absolute/relative frequencies and continuous variables were reported as median/IQR. To study our primary outcome of whether there was an increased risk of ipsilateral axillary recurrence following the omission of axillary surgery at the time of CWR resection, a chi-square test was used to compare the categorical variables between patients with axillary surgery and those without, with *p* < 0.05 defined as being statistically significant. SAS statistical software (v9.4) was used for the analysis.

The study was approved by the SingHealth Centralised Institutional Review Board (CIRB Ref: 2019/2419), and patients’ informed consents were waived by the ethics committee.

## 3. Results

Of 26,754 stage 0-III female breast cancer patients treated during this period, 14,546 (54.4%) underwent mastectomy. Of the patients who underwent mastectomy, 402 (2.76%) developed invasive CWRs. Of these 402 patients, 199 were excluded because of concomitant regional and/or distant metastasis, bilateral breast cancers, no operation for their CWR, incomplete data and clinical or radiological suspicion of ipsilateral axillary nodal involvement at CWR diagnosis in 76, 62, 31, 30 and 9 patients, respectively, leaving 194 (1.3%) patients eligible for analysis.

Of these 194 patients with CWRs, the median age at diagnosis of the primary cancer was 49.5 (IQR 42.3–60) years old. The median primary invasive cancer size was 21.0 (IQR 15.0–35.0) mm. In this cohort, 71.6% had no breast reconstruction and 66.5% had ALND during primary surgery. Of the invasive cancers with a known receptor status, 82.9% had ER positivity and 72.7% were HER2 negative. Among these 194 patients with CWRs, 8 (4.1%), 5 (2.6%) and 181 (93.3%) patients had ALND, SLNB and no axillary operation for their CWR, respectively. The patients with no axillary operation for their CWR were statistically associated with an older age at diagnosis of primary cancer (*p* = 0.022) and a lower incidence of DCIS as primary cancer diagnosis (*p* = 0.007) (Table 1).

The median age at CWR diagnosis was 56.0 years old (IQR 47.0–67.0), with a median CWR size of 14.0 mm (IQR 10.0–23.0). The mean time from primary cancer to CWR was 62.8 months (range:2–298). The CWR histology was similar to that of primary cancer, with ER positivity and HER2 negativity in 83.2% and 78.4% of patients, respectively.

At the diagnosis of CWR, 34 (17.5%) patients underwent axillary ultrasound, which revealed normal findings. Of these 34 patients, 1 patient had SLNB, which revealed no metastatic nodal disease. The remaining 33 patients had no axillary restaging operation, and only 1/33 (3.0%) patients subsequently developed ipsilateral axillary and distant recurrence at the 65 month follow-up period.

There were no statistical differences in the CWR features between the patients with or without axillary operation for their CWR (Table 2). Of the 13 patients who underwent axillary operation, only 1 (7.7%) patient had nodal metastasis. This patient did not have any axillary ultrasound, although the staging CT revealed no ipsilateral axillary lymphadenopathy. This patient underwent an ALND and remained free of second recurrence.

After a median follow-up of 59.5 months (IQR 27.3–105), there were 15 (7.7%) local, 8 (4.1%) regional and 46 (23.7%) distant second recurrences, respectively. Of these recurrences, there were 11 (5.7%) patients with involvement of the ipsilateral axilla lymph nodes. In the group of patients with an omission of axillary restaging operation, the rate of ipsilateral axillary lymph node recurrence was low at 6.1%, which occurred at a median follow-up time of 50.0 months (IQR 11.0–74.0). Second recurrences (*p* = 0.061) and subsequent ipsilateral axillary recurrences (*p* = 0.768) were not statistically different between patients with and without axillary restaging operation at CWR (Table 2).

## 4. Discussion

In patients with CWRs and no evidence or suspicion of concomitant metastasis elsewhere, the omission of axillary restaging was statistically associated, at primary cancer diagnosis, with an older age and a lower incidence of DCIS as diagnosis, as compared to the group of patients with axillary restaging. The omission of the axillary restaging operation did not show a statistically increased risk of subsequent ipsilateral axillary or second recurrences on long-term follow-up. This study has one of the largest series of patients to investigate the oncological outcomes of the omission of the axillary restaging operation in patients with CWRs.

Our incidence of CWRs was comparable with the literature, which also reported a very low incidence of isolated CWRs after mastectomy, at 1–3% [12,13,14], which also explains the sparse data on the axillary management of this group of patients. In contrast, local recurrences following breast conservation have been reported more frequently, hence there are more available data on the axillary management of this group of patients during recurrence [15,16]. Based largely on the literature in patients with prior breast conservation and local recurrence, axillary restaging, in the form of SLNB, was advocated, since it could potentially result in a change in management [15,17,18].

However, it remains unclear if these data, obtained from studies that predominantly contain patients with breast-conserving surgery, could be extrapolated to patients with prior mastectomy, since there were some differences between these groups of patients. Firstly, in patients who had undergone a mastectomy, almost all would have received some form of axillary surgery previously, unlike patients with breast conservation, whereby some could have omitted axillary staging, especially when the primary tumour was ductal carcinoma in situ (DCIS), since the risk of nodal involvement in DCIS is low [19]. This axillary surgery would cause scarring and fibrosis, hence resulting in an aberrant lymphatic drainage. Secondly, patients who underwent mastectomy usually tended to have a larger tumour size compared to patients with breast conservation [20]. This could also be associated with a higher incidence of nodal disease [21,22] and ALND, which could again disrupt lymphatic drainage. These risk factors for aberrant lymphatic drainage in patients who had mastectomy, coupled with the lack of breast tissue after mastectomy, made SLNB for restaging at recurrence for this group of patients more technically challenging. A repeat axillary surgery could also be associated with a higher incidence of complications such as nerve injury and lymphedema, etc.

While there was generally a lower SLNB identification rate of 65.3% (17) and a higher incidence of aberrant lymphatic drainage of 25.7–40% [17,23] in the recurrent setting, it could be harder to achieve a successful SLNB after mastectomy than after breast conservation. This was because the rate of aberrant lymphatic drainage after mastectomy was higher, and had been reported to be as high as 77% [17]. As a result, a lower success rate of axillary SLNB of 63.6–80% [10,24] and 76.9% [10] was reported in patients after mastectomy with prior SLNB or ALND, respectively, in contrast to a 94.7% [25] success rate in patients with prior breast conservation and SLNB. In a specialised centre, however, a successful ipsilateral axillary SLNB as high as 93.4% was reported in patients with CWRs and no prior ALND [26].

Despite the lower SLNB identification rate and the higher rate of aberrant lymphatic drainage in the recurrent setting, SLNB has been reported to be accurate with a low false negative rate of 0.2% [17]. The subsequent ipsilateral axillary recurrence rate was also low at 1.0% [27] in patients with non-metastatic SLNB at their local recurrence, after a median follow-up of 4.7 years. While these were promising results, they were based on a cohort consisting predominantly of patients with breast conservation and few patients with mastectomy, hence their applicability to patients with CWRs remained questionable.

While SLNB could be performed successfully in the recurrent setting, albeit with a lower success rate in patients after mastectomy, it remained controversial if axillary restaging should be performed at all for patients with CWRs and a clinically nodal negative axillary status. Proponents of SLNB would argue that SLNB was needed as it could alter adjuvant treatment, though there were sparse data in this area for patients with CWRs. However, this role of SLNB in decision making for adjuvant treatment may not be absolutely warranted since the receipt of these treatment, such as chemotherapy and hormonal therapy, etc., were more reliant on the tumour molecular subtype [28] and/or genomic tests. Radiotherapy would usually be recommended for patients with CWRs unless it had been previously given [29].

In our study, only one patient with normal axillary ultrasound and no axillary restaging developed subsequent ipsilateral axillary and distant recurrence. However, this occurred more than 5 years after the CWR operation, suggesting that this ipsilateral axillary recurrence may not have been present at the time of CWR diagnosis. Conversely, the rest of the patients with normal axillary ultrasound at CWR diagnosis and omission of the axillary restaging operation had no evidence of subsequent ipsilateral axillary recurrence. This could indicate the role of axillary ultrasound as an adjunct investigation when deciding on the need for an axillary restaging operation. The axillary ultrasound in the recurrence setting was reported to have a sensitivity and specificity of 92.7% and 93.9%, respectively [30]. It also has a higher sensitivity than clinical examination in the detection of subclinical nodal burden [31]. In cases with abnormal axillary imaging, an axillary restaging operation or a percutaneous biopsy could be performed for patients, while an axillary restaging operation could be safely omitted in patients with normal axilla imaging. In our study, however, only 17.5% had an axillary ultrasound, hence the adjunct role of axillary ultrasound in this setting could be further explored in future studies.

In our study, the omission of axillary surgery was also associated with an older age at primary cancer diagnosis. This finding may be attributed to the respective treating surgeon’s perceptions of young age [32] being an adverse prognostic factor, hence resulting in a higher likelihood of axillary restaging surgery in these subgroups. However, the rate of nodal involvement in the group of patients who had an axillary restaging operation in our study was low.

It was also not surprising in our study that patients having a diagnosis of DCIS as primary cancer diagnosis were more likely to have an axillary restaging operation at the time of CWR. This could be explained by a higher incidence of SLNB being performed at primary operation for DCIS; hence, there were fewer perceived technical difficulties compared to prior ALND which has a higher incidence of aberrant lymphatic drainage.

The strengths of this study included the fact that it is currently one of the largest series, to the best of our knowledge, which has investigated the effect of the omission of axillary restaging surgery in patients with seemingly isolated CWRs at diagnosis on subsequent second recurrence. Being a multicentre study, the results of this study were also more applicable to a larger population. There was also a long follow-up of patients’ recurrence outcomes.

The limitations of the study included the fact that because it was a retrospective study, there may have been selection bias for treatment. However, the treatment variables were not statistically significant between the two comparison groups. Nonetheless, there could also be unmeasured confounders that can affect the results. For instance, patient comorbidities, surgeon expertise and specific tumour characteristics might influence the outcomes, but were not accounted for. The patients who underwent an axillary restaging operation in our study, as a comparison group, were of a small sample size. This imbalance can affect the statistical power of the study and the reliability of comparative results. Nonetheless, this study provided a sizable number of patients with CWRs and no axillary surgery with long-term follow-up data. Certain data points were not available for all patients (e.g., invasive tumour size, hormone receptor status, and HER2 status). These missing data could bias results and limit the generalizability of the findings. As a result, this study’s findings have to be applied cautiously to patients after considering their individual characteristics. Ideally, a randomised controlled trial or a prospective study with sample size calculation would provide the best evidence to determine the oncological safety of the omission of an axillary restaging operation in patients with seemingly isolated CWRs, since it could minimise confounding effects and avoid an imbalance in the comparison group. However, this may not be possible to achieve, given the low incidence of isolated CWRs.

## 5. Conclusions

In patients with CWRs after mastectomy and having no evidence or suspicion of metastasis elsewhere, the omission of an axillary restaging operation was not statistically associated with an increased risk of subsequent ipsilateral axillary or second recurrences on long-term follow-up. Axillary ultrasound could be used as an adjunct for decision making on the omission of an axillary restaging operation. This finding could provide more data on this sparse topic and further define the current guidelines on the axillary management of patients with isolated CWRs.

## Figures and Tables

**Table 1:** Characteristics of the primary cancer in patients with isolated chest wall recurrences (CWRs).

Clinical Features of Primary Cancer N = 194	Patients with No Axillary Surgery at CWR N = 181/(%)	Patients with Sentinel Lymph Node Biopsy and/or Axillary Lymph Node Dissection at CWR N = 13/(%)	*p* Value
Age (years)			0.022
<50	86 (47.5)	11 (84.6)	
≥50	95 (52.5)	2 (15.4)	
Reconstruction			0.067
Yes	47 (26.1)	7 (53.8)	
No	133 (73.9)	6 (46.2)	
Unknown	1	0	
Axillary surgery			0.129
Not done	3 (1.7)	1 (7.7)	
Sentinel lymph node biopsy	53 (29.6)	6 (46.2)	
Axillary lymph node dissection	123 (68.7)	6 (46.2)	
Unknown	2	0	
Tumour histology			0.007
Ductal carcinoma in situ	12 (6.6)	4 (30.8)	
Invasive ductal	143 (79.0)	7 (53.8)	
Invasive lobular	15 (8.3)	0 (0)	
Others	11 (6.1)	2 (15.4)	
Pathological features			
Invasive tumour size (mm) ^1^			0.585
≤20	73 (46.5)	5 (62.5)	
>20 to ≤50	74 (47.1)	3 (37.5)	
>50	10 (6.4)	0 (0)	
Not applicable/Unknown	24	5	
Grade of invasive cancer			0.703
I	20 (12.8)	2 (22.2)	
II	84 (53.8)	4 (44.4)	
III	52 (33.3)	3 (33.3)	
Not applicable/Unknown	25	4	
Oestrogen receptor (ER) ^2^			1.000
Positive	134 (82.7)	7 (87.5)	
Negative	28 (17.3)	1 (12.5)	
Not applicable/Unknown	19	5	
Progesterone receptor (PR) ^2^			0.610
Positive	115 (72.8)	7 (87.5)	
Negative	43 (27.2)	1 (12.5)	
Not applicable/Unknown	23	5	
Human epidermal growth factor receptor 2 (HER2) ^2^			1.000
Positive	37 (27.0)	2 (33.3)	
Negative	100 (73.0)	4 (66.7)	
Not applicable/Unknown	44	7	
Nodal status			0.063
pN0	109 (62.6)	12 (92.3)	
pN+	65 (37.4)	1 (7.7)	
Unknown	7	0	
Chemotherapy ^2^			0.708
Yes	77 (45.6)	3 (33.3)	
No	92 (54.4)	6 (66.7)	
Not applicable/Unknown	12	4	
Radiotherapy			0.203
Yes	32 (17.7)	0 (0.0)	
No	149 (82.3)	13 (100.0)	
Hormonal therapy			0.636
Yes	130 (71.8)	8 (61.5)	
No	51 (28.2)	5 (38.5)	

^1^ If multifocal/centric disease was present, the size measurement was based on the largest lesion. ^2^ Invasive cancer only.

**Table 2:** Characteristics of the chest wall recurrences (CWRs).

Clinical Features N = 194	Patients with No Axillary Surgery at CWR N = 181/(%)	Patients with Sentinel Lymph Node Biopsy and/or Axillary Lymph Node Dissection at CWR N = 13/(%)	*p* Value
Age (years) at recurrence			0.486
<50	59 (32.6%)	6 (46.2%)	
≥50	122 (67.4%)	7 (53.8%)	
Pathological features of the recurrences			0.072
Invasive tumour size (mm) ^1^			
≤20	72 (72.0)	3 (37.5%)	
>20 to ≤50	25 (25.0)	5 (62.5%)	
>50	3 (3.0)	0 (0)	
Not available	81	5	
Tumour histology			0.078
Invasive ductal	116 (80.0)	8 (66.7)	
Invasive lobular	12 (8.3)	0 (0.0)	
Others	17 (11.7)	4 (33.3)	
Unknown	36	1	
Grade			0.387
I	11 (10.2)	0 (0)	
II	56 (51.9)	6 (75.0)	
III	41 (38.0)	2 (25.0)	
Not available	73	5	
Oestrogen receptor (ER)			1.000
Positive	138 (83.1)	11 (84.6)	
Negative	28 (16.9)	2 (15.4)	
Not available	15	0	
Progesterone receptor (PR)			0.461
Positive	95 (59.7)	9 (75.0)	
Negative	64 (40.3)	3 (25.0)	
Not available	22	1	
Human epidermal growth factor receptor 2 (HER2)			0.923
Positive	30 (21.1)	3 (27.3)	
Negative	112 (78.9)	8 (72.7)	
Not available	39	2	
Pathological nodal involvement ^2^			-
Yes	-	1 (8.3)	
No	-	11 (91.7)	
Unknown		1	
Chemotherapy			0.237
Yes	43 (32.1)	6 (54.5)	
No	91 (67.9)	5 (45.5)	
Unknown	47	2	
Radiotherapy			0.291
Yes	120 (66.3)	11 (84.6)	
No	61 (33.7)	2 (15.4)	
Hormonal therapy			0.841
Yes	132 (78.1)	11 (84.6)	
No	37 (21.9)	2 (15.4)	
Not available/not applicable	12	0	
Targeted HER2 therapy			1.000
Yes	13 (50.0)	2 (66.7)	
No	13 (50.0)	1 (33.3)	
Unknown/not applicable	155	10	
Ipsilateral axillary recurrence as subsequent recurrence			0.768
Yes	11 (6.1)	0 (0.0)	
No	170 (93.9)	13 (100.0)	
Second recurrence			0.061
No	113 (62.4)	12 (92.3)	
Yes	68 (37.6)	1 (7.7)	

^1^ Based on the largest invasive tumour size if multiple lesions present. ^2^ Based on the patients who underwent axillary surgery.

## Data Availability

The data can be made available from the corresponding author, upon request.
